# Effect of ageing and immersion in different beverages on properties of denture lining materials

**DOI:** 10.1590/S1678-77572010000400009

**Published:** 2010

**Authors:** Vanessa M. F. LEITE, Marina X. PISANI, Helena F. O. PARANHOS, Raphael F. SOUZA, Cláudia H. SILVA-LOVATO

**Affiliations:** 1 DDS, Graduate student, Department of Dental Materials and Prosthodontics, Ribeirão Preto Dental School, University of São Paulo, Ribeirão Preto, SP, Brazil.; 2 DDS, MSc, Graduate student, Department of Dental Materials and Prosthodontics, Ribeirão Preto Dental School, University of São Paulo, Ribeirão Preto, SP, Brazil.; 3 DDS, MSc, PhD, Titular Professor, Department of Dental Materials and Prosthodontics, Ribeirão Preto Dental School, University of São Paulo, Ribeirão Preto, SP, Brazil.; 4 DDS, MSc, PhD, Assistant Professor, Department of Dental Materials and Prosthodontics, Ribeirão Preto Dental School, University of São Paulo, Ribeirão Preto, SP, Brazil.; 5 DDS, MSc, PhD, Associate Professor, Department of Dental Materials and Prosthodontics, Ribeirão Preto Dental School, University of São Paulo, Ribeirão Preto, SP, Brazil.

**Keywords:** Denture lining, Technical processing, Thermal cycling, Immersion colorants, properties

## Abstract

**Objectives:**

To evaluate the color stability and hardness of two denture liners obtained by direct and indirect techniques, after thermal cycling and immersion in beverages that can cause staining of teeth.

**Material and Methods:**

Seventy disc-shaped specimens (18 x 3 mm) processed by direct (DT) and indirect techniques (IT) were made from elite soft (n=35) and Kooliner (n=35) denture liners. For each material and technique, 10 specimens were subjected to thermal cycling (3,000 cycles) and 25 specimens were stored in water, coffee, tea, soda and red wine for 36 days. The values of color change, Shore A hardness (elite soft) and Knoop hardness (Kooliner) were obtained. The data were subjected to ANOVA, Tukey’s multiple-comparison test, and Kruskal-Wallis test (P<0.05).

**Results:**

The thermal cycling promoted a decrease on hardness of Kooliner regardless of the technique used (Initial: 9.09±1.61; Thermal cycling: 7.77±1.47) and promoted an increase in the hardness in the DT for elite Soft (Initial: 40.63±1.07; Thermal cycling: 43.53±1.03); hardness of Kooliner (DT: 8.76±0.95; IT: 7.70±1.62) and elite Soft (DT: 42.75±1.54; IT=39.30±2.31) from the DT suffered an increase after the immersion in the beverages. The thermal cycling promoted color change only for Kooliner in the IT. Immersion in the beverages did not promote color change for elite in both techniques. The control group of the DT of Kooliner showed a significant color change. Wine and coffee produced the greatest color change in the DT only for elite Soft when compared to the other beverages.

**Conclusion:**

The three variation factors promoted alteration on hardness and color of the tested denture lining materials.

## INTRODUCTION

The development and use of denture liners have been essential for providing an even distribution of functional load on the denture bearing area, avoiding local stress concentrations and improving comfort to the patients^5^.

The denture liners can be chemically and thermally processed, by direct and indirect techniques, respectively. The liners processed by the indirect technique show higher degree of polymerization, an increase in the polymerization shrinkage, therefore, a lower dimensional stability^23,26,27^. The direct technique promotes a lesser degree of polymerization; the material obtained has a potential for tissue irritation due to the higher concentration of monomer non-reacted and less color stability in the presence of tertiary amines. However, this technique promotes a decrease in the degree of polymerization contraction and better dimensional stability^25,27^.

The ideal resilient liner should present characteristics such as permanent resilience or resilience over an extended period of time, capability of forming a strong bond with acrylic or other rigid denture-base materials, dimensional stability, adequate tear resistance and permanence, including color stability, shelf line and freedom from unwanted absorption of flavors, odors or bacterial growth^7^. In the meantime, the denture liner materials still show instability with relation to these proprieties. Some of the liners are not stable in aqueous environment such as the oral cavity. Due to a threefold sequence of events consisting of loss of ethanol, water absorption and loss of plasticizer, lining materials tend to harden hence limiting their usefulness.

A simple manner to measure the elasticity module or the softness of an elastomer is using the hardness test based on the measurement of resistance to penetration of an indenter when a force is applied. However, the maintenance of hardness is one of the most difficult factors in the use of liners, since most of them are not stable in water and in the oral cavity. Thermal effects from ingestion of hot and cold food and drinks may also have a deleterious consequence^5,9^.

Color stability is a required characteristic of denture base polymers, whether hard acrylics or soft lining materials^2,32^. There are evidences that beverages such as tea, coffee and wine significantly increase developing stains on both denture base polymer and soft lining materials^5^. The change in color can be considered as an indicator of aging or damage of a material. Some authors have investigated the changes in color of soft liners through its aging and noticed substantial changes in their properties, particularly the acrylic liners, caused by water sorption or solubilization^15,19^.

This study was undertaken to establish the changes in the color and hardness of two denture liners, according to the following factors: direct and indirect processing techniques, artificial accelerated aging by thermal cycling, and exposure to different beverages that can cause staining of teeth.

## MATERIAL AND METHODS

Two chemically and structurally different, commercially available lining materials were used: an acrylic resin or polymethylmethacrylate-based material (Kooliner; GC America Inc., Alsip, IL, USA) and a silicone-based material (elite Soft relining; Kettenbach, Germany). Kooliner is a powder-liquid systems and elite soft is a two-component paste.

### Indirect Polymerization technique

Five circular stainless steel matrices (18 mm x 3 mm) were included in flasks with plaster rock type III (Herodent, Vigodent SA Ind. e Com., Rio de Janeiro, RJ, Brazil) and silicon for inclusion (Zetalabor, Zermack, Badia, Polesine, Italy). After polymerization of the inclusion materials, the matrices were deflasked and the denture liners were introduced into the moulds. The flasks were stored under the press (PM-2000, Techno Machine Ltd., Vinhedo, SP, Brazil) for approximately 10 min, and pressure was applied slowly and gradually, reaching up to 1.25 (tons). Then they were placed in a polymerase thermostatically controlled apparatus (Promeco, São Paulo, Brazil) with water at a temperature of 40-45ºC for 10 min, following the manufacturers’ recommendations for each material.

The specimens were removed from the flasks and finished. For Kooliner, it was conducted with tungsten carbide drills, number 1572 (edenta AG, Switzerland). For elite Soft, the material excesses were cut out with a sharp knife. Thirty-five specimens of each material were obtained. Ten of them underwent thermal cycling and the other 25 were subjected to immersion in the test beverages.

### Direct polymerization technique

The same moulds obtained for the indirect polymerization were used for the direct technique. In this technique, the flasks were placed on the press at 1.25 tons and the polymerization was conducted for 10 min at room temperature following the manufacturers’ instructions. After this, the same finishing process was used. Thirty-five specimens of each material were obtained. Ten of them underwent thermal cycling and the other 25 were immersed in the test beverages.

### Hardness

For elite Soft, the Shore A durometer (Instrument and Manufacturing Co. Inc, Freeport, NY, USA) was used, while for Kooliner Knoop hardness was measured using the Shimadzeu microhardness tester (Shimadzeu, model-HMV-2, Tokyo, Japan)^1^. All specimens were subjected to these tests immediately after finishing, after thermal cycles and after immersion in the test beverages.

### Color change

The color change was measured using a portable spectrocolorimeter (Color Guide 45/0; BYK-Gardner, Latin America, Santo André, SP, Brazil). This instrument was used to quantify the values of color change (∆E) from the data obtained before and after application of the factors described. The results after each experimental condition were compared to those originally obtained for each specimen.

### Thermal cycling

After analysis of the initial color and hardness, 10 specimens of each material for each processing technique underwent accelerate ageing through thermal cycles where the specimens were subjected for 3,000 cycles at temperatures ranging from 5 to 55ºC with a 60-s dwell time. The number of cycles was used to simulate complete denture use for approximately 2 years^15^. After this procedure, the specimens were again subjected to hardness and color analysis.

### Immersion in food the beverages

After the initial color and hardness analysis, 25 specimens of each material for each technical processing were stored in different beverages that can cause staining of teeth at 37ºC. The specimens were stored individually in containers with 20 mL of solution, which was replaced daily. Controls (n =5) were immersed in distilled water (Solution 1, or S1). The drinks used were: S2 (n=5), soluble coffee (Nescafé Original, Nestlé, São Paulo, SP, Brazil), prepared according to the manufacturer’s directions; S3 (n=5), solution of tea (Lipton Ice Tea); S4 (n=5), solution of soda (Coca-cola) and S5 (n=5), red wine (Almadén Gamay Rose Red). Guler, et al.^11^ (2005) described that coffee consumption (1 dose) lasts 15 min, given that 3.2 doses are consumed daily by regular coffee drinkers. Thus, the 24-h storage simulate 1 month of regular coffee drinking. In this study, the same immersion time for coffee was used in order to facilitate comparisons. Thus, the immersion period lasted 36 days, which represents 3 years of consumption. At the end of this period, all the specimens were again subjected to hardness tests and color change.

The data were subjected to statistical analysis by ANOVA, Tukey’s multiple-comparison test and Kruskal Wallis test. All data analyses were performed at 0.05 level of significance.

## RESULTS

### Effect of Thermal Cycling on Surface Hardness

Means of Knoop hardness of Kooliner specimens before and after the thermal cycling for both techniques are listed in Table 1. The lowest hardness value was obtained after thermal cycling (P<0.05).

**Tabela 1:** Knoop hardness means (standard deviation) of Kooliner according to the techniques and times (before and after thermal cycling)

**Techniques**	**Before**	**After**	**Means**
			
Direct	8.35 (1.42)	7.45 (1.07)	7.9 (1.24) ª
Indirect	9.84 (1.80)	8.08 (1.88)	8.96 (1.84) ª
Means	9.09 (1.61) ^A^	7.76 (1.47) ^B^	

Different superscripted uppercase letters indicate statistically different means within each column; different superscript lowercase letters indicate statistically different means within each row.

The technique did not influence the hardness. For Elite Soft, there were significant differences in hardness between the times and techniques. Mean of Shore A hardness of elite Soft are given in Table 2. There was an increase of the hardness for the direct technique after thermal cycling and this increase was significantly higher than that of the indirect technique.

**Tabela 2:** Shore A hardness means (standard deviation) of Elite Soft according to the techniques and times (before and after thermal cycling)

**Techniques**	**Before**	**After**
		
Direct	40.63 (1.07) ^Aa^	43.53 (1.03) ^Ba^
Indirect	41.93 (0.91) ^Aa^	42.13 (1.33) ^Ab^

Different superscripted uppercase letters indicate statistically different means within each column; different superscripted lowercase letters indicate statistically different means within each row.

### Effect of Immersion in Food Colorants on Surface Hardness

**Figure 1:**
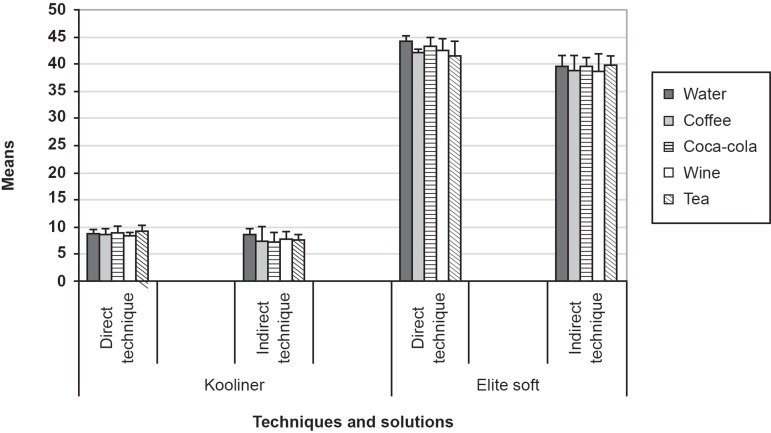
Means and standard deviations of Kooliner and Elite soft hardness: techniques and beverages

For both materials (Kooliner and elite Soft), there was significant variation for the technique (Figure 1). After immersion in the beverages, a more considerable increase of the hardness was noticed for the direct technique than for the indirect technique.

### Effect of Thermal Cycling on Color change

There were significant variations in the material x technique interaction. Kooliner showed higher color change than Elite Soft only in the indirect technique, with no color change differences when comparing the direct and indirect techniques (Table 3).

**Tabela 3:** Color change means (standard deviation) of the materials obtained by the direct and indirect techniques

	**Direct technique**	**Indirect technique**
		
Kooliner	3.78 (1.06) ^Aa^	5.56 (1.64) ^Aa^
Elite Soft	3.86 (1.46) ^Aa^	2.66 (0.85) ^Ab^

Different superscripted uppercase letters indicate statistically different means within each column; different superscripted lowercase letters indicate statistically different means within each row

### Effect of Immersion in the Beverages on Color Change

There were significant differences between the color change means of the materials obtained for the direct and indirect techniques after immersion in different beverages (Table 4).

**Tabela 4:** Comparison of color change means (standard deviation) of the materials on interaction of techniques and beverages

**Beverages**	**Elite soft**		**Kooliner**	
	**Direct technique**	**Indirect technique**	**Direct technique**	**Indirect technique**
Water	1.81 (1.13)^Aa^	1.49 (0.88)^Aa^	4.11 (1.06)^Aa^	1.97 (0.61)^Ba^
Wine	18.25 (1.0)^Ab^	15.98 (3.37)^Ab^	3.64 (0.99)^Aa^	2.40 (0.42)^Aa^
Coca-cola	1.34 (0.60)^Aa^	2.44 (1.28)^Aa^	2.89 (1.83)^Aa^	1.61 (0.40)^Aa^
Coffee	5.06 (1.41)^Ac^	4.32 (0.73)^Aa^	4.05 (0.92)^Aa^	2.99 (0.57)^Aa^
Tea	3.15 (1.38) ^Aac^	4.75 (1.51)^Aa^	2.97 (1.23)^Aa^	2.04 (1.21)^Aa^

Different superscripted uppercase letters indicate statistically different means within each column; different superscripted lowercase letters indicate statistically different means within each row.

Elite Soft did not present any significant differences between the direct and indirect techniques. Kooliner only presented differences between the techniques in the control group with higher values of color change for the direct technique. For beverages, Kooliner did not present any significant color change when immersed in the beverages; elite Soft presented color change when immersed in the different colorants, and the wine was the solution that caused the highest value of color change. Generally, elite Soft (m=5.85±1.33) showed a higher color variation than Kooliner (m=2.86±0.92) after immersion in the beverages. However, this difference was not significant (*P* >0.05).

## DISCUSSION

There are many types of denture lining materials used for prosthetic purposes. Acrylic soft resins and silicone rubber are often preferred, although there are also the hard acrylic resins, but with greater resilience when compared to conventional acrylic resins. The acrylic resin materials are acrylic copolymers to which plasticizers may be added. These materials may absorb water, swell, and harden because of plasticizer leaching^16^. For these reasons, their intraoral efficacy is shortlived. Silicone rubber material is composed of dimethylsiloxane polymer, which is a viscous liquid, cross-linked to provide good elastic properties. These materials excel in their resiliencies and in their initial resistance to water sorption^29^.

Direct auto polymerizing reline resins, whether hard or soft, are currently being used in dentistry as interim liners for immediate dentures and for interim dentures until a definitive denture is completed. These are called chair side liners because they allow the dentist to reline a removable denture directly in the patient’s mouth. The procedure is faster than the indirect polymerization technique, in which a hot water bath was used^8^ at a temperature of 4045ºC to promote the material’s final polymerization. However, the direct autopolymerizing reline resins can cause a chemical burn on the mucosa, poor color stability, greater porosity, malodor, sluggish flow, tissue surface deficits and poor bonding, in addition to high polymerization temperatures. Indirect polymerization for heat-cured denture liners is also recommended by the manufacturers of the two materials used in this study (Kooliner and elite Soft), with the aim of relining the removable denture out of the patient’s mouth.

The color change is clinically important and is one of the criteria providing information on the serviceability of these materials^5^. In the present study, the color test was made according to ADA specification number 17^6^.

Johnston and Kao^15^ (1989) observed that if *Δ* e is less than 1, this chromatic value is deemed to be very small and between 1 and 2, hence the situation is clinically acceptable. However, there is some controversy in the literature with regard to which *Δ* e values can actually be seen by the naked eye or are clinically relevant. Some investigators assume that *Δ* es from 2 to 3 are just visible; Ma, et al.^18^ (1997) state a *Δ* e value of 1 as a distinguishable value. Ruyter, et al.^30^ (1987) describe discoloration of *Δ* e*>* 3.3 as no longer clinically acceptable.

The change in color of dental restorative materials may be caused by stain accumulation, dehydration, water sorption, leakage, poor bonding and surface roughness, wear or chemical degradation, oxidation of the reacted carbon-carbon double bonds that produces colored peroxide compounds, and continuing formation of the colored degradation products. The color changes in soft denture liners are attributed to changes in the colorants used that cause a color change in the elastomer, or both. Some colorants or elastomer may be affected by high humidity or warmer temperature in the aging chamber. However the mechanism of color change is not known exactly, but it can be estimated despite knowing how aging changes the physical and mechanical properties of soft denture liners^10,21,24,31^.

The proper functioning of soft liners depends to a great extent on their mechanical properties. As with many polymers, these materials are affected by ageing^8,16^. An ageing device was used to subject samples to both visible and UV light and distilled water sprays to simulate aging. Although the oral environment is more complex, this simulated aging treatment is useful for comparing different materials^31^. Due to the lack of specifications for determining how much thermal cycling the soft liners should be subjected to, in this study, the number of daily meals was used as reference. A person usually has six meals a day, 6 thermal shocks daily. In this study, a thermal cycling regimen with 3,000 cycles was used because it corresponds to approximately 2 years of simulated use of the soft liner. Thermal cycling promoted a color change only for Kooliner in the indirect technique, this assertion is in agreement with Jin, et al.^14^ (2003) who used denture cleansers as a simulative aging agent for soft liners and they found that silicone soft liners were more stable in surface roughness and in color change than the acrylic soft liners. In a similar study, Shotwell, et al.^32^ (1992) concluded that there is a variation range between materials that have different composition. Anil, et al.^3^ (1999) investigated the color stability of heat polymerized and autopolymerized soft denture liners and found that the autopolymerized ones presented the greatest color change, which is not in agreement with this study.

Changes in the molecular structure of polymers as a result of the aging process have been attributed to the following factors: scission of the polymer chains by UV light; oxygen cross linking; leaching of plasticizers; water sorption^33^.

There is evidence that beverages such as tea, coffee and wine increase significantly stain development on denture base polymers and soft denture liners^5^. Liquid intake specially causes surface staining, which is the reason why we selected such drinks for this study. There are many causes for staining of teeth such as consumption of certain beverages, such as tea, coffee, wine and coca-cola, and smoking. Some of the authors claim that tea is more staining than smoking on silicone based polymers^7^. In the present study, there was no significant difference among the beverages, except for the wine and coffee that produced the greatest color change in elite Soft auto polymerizing soft liner.

The immersion in the selected beverages in this study did not promote color change for the silicone based elite Soft, but changes in color were observed in the direct technique for Kooliner. Water or saliva can be absorbed into the material, and plasticizers or other constituents of the lining material can be leached^17,20,28^. Canay, et al.^5^ (1999) evaluated the color changes of soft lining materials in beverages and reported that polymethyl/ethyl methacrylate-based soft denture liners produced slightly greater color changes than the siliconebased liners. Polymethyl/ethyl methacrylate polymer Kooliner is hydrophilic, attracting water soluble dyes to the surface of the lining material as a result of electrostatic charges^5^. However, silicone-type polymer elite Soft is a hydrophobic and inert nonwettable polymer, thus it could be more resistant to color change^31^.

From a theoretical standpoint, liners should be in order to distribute functional stresses on the residual ridge, and they should also absorb energy during mastication to reduce transmission of that energy to the mucosa^34^. Some soft liners can deform permanently, and although a small amount of deformation could be beneficial, hence allowing the liner to adapt to changes in the natural tissues, any significant change can cause instability to a denture^13^. During clinical use, resilient lining materials undergo changes in hardness, thus rendering them ineffective^22^. In the present study the thermal cycling altered the physical property of hardness. An increase in this property was noted for the direct technique of elite Soft and a decrease for Kooliner. Hekimoglu and Anil^12^ (1999) observed that the thermal cycling promoted an increase in the hardness and in the solubility of the lining materials.The storage effect in the beverages was the hardness increase of the lining materials, which is in agreement with Buudai, et al.^4^ (1995). It is possible to assume that the leaching out of the plasticizer and the absorption of the liquid are responsible for this fact. This study is important because it should prove valuable to clinicians when they are choosing the ideal lining material for the patients, if they are concerned with color stability, polymerization technique and physical properties associated with use, although that this study has some limitations of an *in vitro* study. Further *in vivo* investigations are necessary to compare the actuation and properties of these denture liners in clinical use.

## CONCLUSIONS

Within the limitations of this study, the following conclusions were drawn: Regarding hardness, the thermal cycling promoted a decrease in the hardness of Kooliner whatever the technique used and promoted an increase in the hardness of elite Soft from the direct technique; the hardness of Kooliner and elite Soft from the direct technique suffered an increase after immersion in the beverages. Regarding color change, thermal cycling promoted color change only for Kooliner from the indirect technique. The immersion in the different beverages did not promote color change for elite in neither of the techniques. Only for the control group in the direct technique of Kooliner there was a significant color change. Wine and coffee produced the greatest color change for elite Soft only in the direct technique when compared to the other beverages.
